# The effect of eave and window modifications on house entry behavior of *Anopheles gambiae*

**DOI:** 10.1186/s13071-025-06887-9

**Published:** 2025-07-03

**Authors:** Jeroen Spitzen, Martin J. Lankheet, Remco P. M. Pieters, Miracle Gadamika, Ike Phiri, Antoine Cribellier, James G. Logan, Constantianus J. M. Koenraadt, Kamija S. Phiri, Florian T. Muijres, Robert S. McCann

**Affiliations:** 1 Laboratory of Entomology, Department of Plant Sciences, Wageningen University & Research, Wageningen, The Netherlands; 2 Centre for Monitoring of Vectors, Netherlands Institute for Vectors, Invasive Plants and Plant Health, Netherlands Food and Consumer Product Safety Authority, Wageningen, The Netherlands; 3 Experimental Zoology Group, Department of Animal Sciences, Wageningen University & Research, Wageningen, The Netherlands; 4 School of Global and Public Health, Kamuzu University of Health Sciences, Blantyre, Malawi; 5 Malawi University of Business and Applied Sciences, Blantyre, Malawi; 6 London School of Hygiene and Tropical Medicine, London, UK; 7 Center for Vaccine Development and Global Health, University of Maryland School of Medicine, Baltimore, MD USA

**Keywords:** *Anopheles gambiae*, Malaria, Mosquito control, Housing, Insect flight, Videography

## Abstract

**Background:**

* Anopheles gambiae* mosquitoes transmit malaria parasites to humans mostly by biting them indoors at night, entering houses predominantly through ventilation openings such as open eaves and windows. In the study reported here, we studied how flying *An. gambiae* approach and enter a house, and whether barriers to reduce mosquito house entry alter mosquito flight patterns.

**Methods:**

Stereoscopic high-speed videography was used to reconstruct nearly 70,000 three-dimensional tracks of mosquitoes flying around a house during 30 experimental nights, with five combinations of closed or screened eaves and windows (eaves open – windows open; eaves open – windows closed; eaves open – windows screened; eaves closed – windows screened; eaves screened – windows screened).

**Results:**

In this study the eave and window treatments did not affect the number of mosquitoes attracted to the house. In all cases, mosquitoes were most active during the early evening, with lower but sustained activity throughout the night. Most *An. gambiae* mosquitoes approached the house by flying directly towards the eave in an upward sloping path with minimal left–right deviations, and most flight activity near the house was directly in front of the eave. Due to the highly attractive nature of the eave area of the house, window treatments had limited to no effect on the number of house entries when eaves were left open, highlighting the importance of closing or screening eaves to prevent mosquito house entry. For the screened eave treatment, *An. gambiae* spent about 10-fold more time near the eave over the course of the night compared to the time spent near the eave in the open or closed eave treatments. Moreover, these mosquitoes returned multiple times, persistently trying to enter the house. When the eaves were fully closed, mosquitoes ultimately diverted from the eave area towards the screened window, but the initial approach flights remained towards the closed eave.

**Conclusions:**

Taken together, these results demonstrate the tendency of *An. gambiae* to direct house entry toward the eaves, and to only divert to other house entry points as a secondary option. The persistent mosquito flight near screened eaves may provide guidance for the placement of outdoor vector control tools.

**Graphical abstract:**

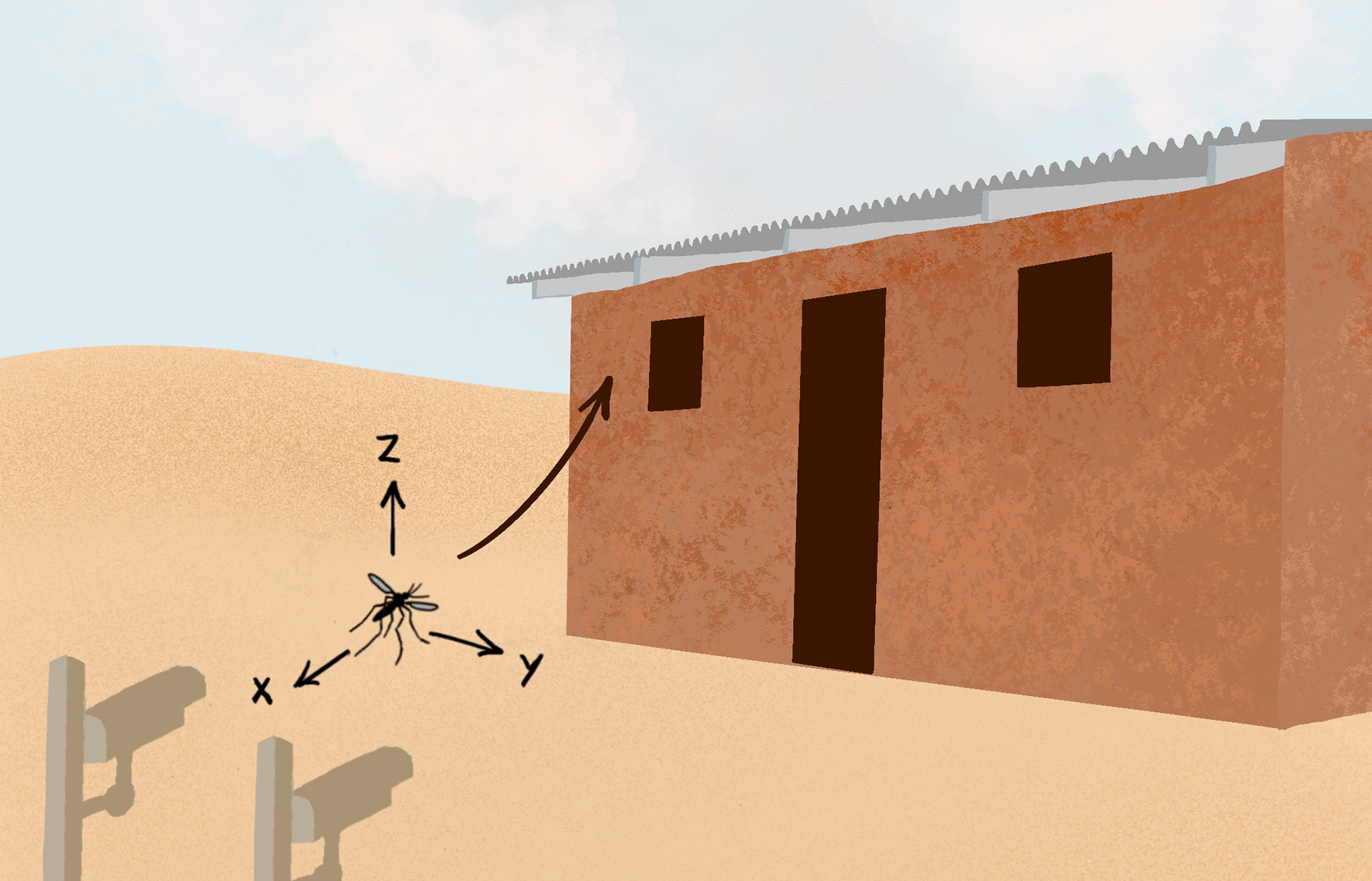

**Supplementary Information:**

The online version contains supplementary material available at 10.1186/s13071-025-06887-9.

## Background

Malaria remains a serious global health challenge, despite the progress made over the past two decades in reducing the malaria burden. From 2000 to 2015, the prevalence of *Plasmodium falciparum* infection in endemic regions of Africa was reduced by 50%, and an estimated 663 million clinical cases of malaria were averted [1]. Vector control, mostly by means of insecticide-treated bednets, contributed to more than 70% of this reduction [1], demonstrating the importance of interrupting mosquito-human contact. However, the progress made up to 2015 has stalled in recent years [2].

The mosquito *Anopheles gambiae* sensu stricto (here after referred to as *An. gambiae*) is one of the main vectors of malaria parasites in Africa. This mosquito species is typically nocturnal, anthropophilic and endophilic, i.e. it prefers to feed from humans indoors at night [3–6]. Hence, a critical component of finding a blood meal host is for the mosquito to find and enter an inhabited house. Navigation towards inhabited houses is most likely triggered by carbon dioxide (CO_2_) levels exceeding background levels [7]. Locating a house, or a house entry point, is presumably based on the integration of perceived CO_2_ levels, volatile host odors and visual cues or contrasts between the house and the landscape [8, 9]. The perception of volatile cues by mosquitoes is affected by odor plume structure, and this, in turn, is affected by habitat characteristics and house design [8, 10]. As such, climate conditions and indoor micro-climate affect the approach to the house and its entry points and the time spent indoors by mosquitoes [10, 11]. It should be noted that the behavioral set of responses for house entry and indoor resting can be distinct from that for actual host finding [12, 13].

Traditional house designs in many malaria-endemic regions of Africa include three types of openings that can potentially be used by mosquitoes as entry points: doors, windows and open eaves. Open eaves, i.e. open gaps usually running the length of the wall where the roof and wall meet but do not join, are particularly important house entry points for *An. gambiae* [12, 14]. Screening eaves has been reported to reduce the number of *An. gambiae* and other malaria vectors in houses [15, 16], even when the windows and doors are left open [17]. Screening windows and doors can reduce the number of *An. gambiae* in houses with closed eaves [10], but not those with open eaves [17].

Traditional house designs, including open eaves and unfinished materials for walls, floors and roofs, are generally associated with an increased risk of malaria parasite infection and malaria cases when compared to improved house designs (closed eaves and finished materials) [18, 19]. In randomized trials, house modifications (some combination of screening or closing off eaves, windows, doors and ceilings) have been associated with reduced malaria parasite prevalence and reduced anemia prevalence [20]. This has led to growing support from policy-makers for structural house modifications as a strategy for malaria vector control [21].

Understanding mosquito flight towards window and eave openings, the time spent near these entry areas and the success rates of finding entry openings provides valuable information for designing mosquito-proof homes [14, 16]. Filming and analyzing mosquito flight tracks around house entry points allows for the collection of detailed information about the flight behavior of mosquitoes when approaching a house and entering houses (e.g. flight trajectories, time spent near windows and eaves, persistence and angle of approach). This fundamental knowledge on mosquito behavior near inhabited houses is essential for designing effective house modifications to prevent mosquito entry [22, 23], and it is also key for the design and optimal placement of other vector control tools used on or near houses, such as eave tubes [24], spatial repellents, odor baited traps or a combined push–pull strategy [25–27].

In the study reported here, we assessed how different modifications of eaves and windows affect the flight dynamics of female *An. gambiae* mosquitoes around the house and, consequently, house entry behavior and rates. Over the course of 240 h of experimental recordings, we captured nearly 70,000 mosquito flight tracks, amounting to approximately 76 h of mosquito flight data. We used these data to quantify the spatial–temporal distribution of mosquitoes flying around the house, their typical house approach and entry behavior and how the eave and window treatments affected these.

## Methods

### Mosquito colony

All mosquitoes used in this study were colony-reared *An. gambiae* (Kisumu strain). Eggs to establish the colony were initially obtained from the Malaria Alert Centre, Blantyre, Malawi, and the colony was maintained in the laboratory facilities at Majete Wildlife Park in Chikwawa District, Malawi. The colony rearing facility was not climate controlled, and the temperature and relative humidity in this facility ranged from 24 °C to 36 °C and from 62% to 85%, respectively. Mosquitoes were allowed to blood-feed twice per week on a human arm, and eggs were distributed over larval rearing trays (46 × 30 × 9 cm) filled with water from a well near the laboratory facility or from a tap at the nearby Kapichira Power Station. Each tray held 300–400 larvae, fed on ground pellets of Marltons koi and pond fish food (Marltons Pet Care Pty Ltd, Westmead, South Africa). Pupae were collected daily and placed in cages for emergence to adults. All cages with adult mosquitoes were provided a 10% sucrose solution via a piece of soaked cotton wool. Cages with experimental mosquitoes were not provided with a blood meal prior to the experiments.

### Experimental set-up

**Fig. 1:**
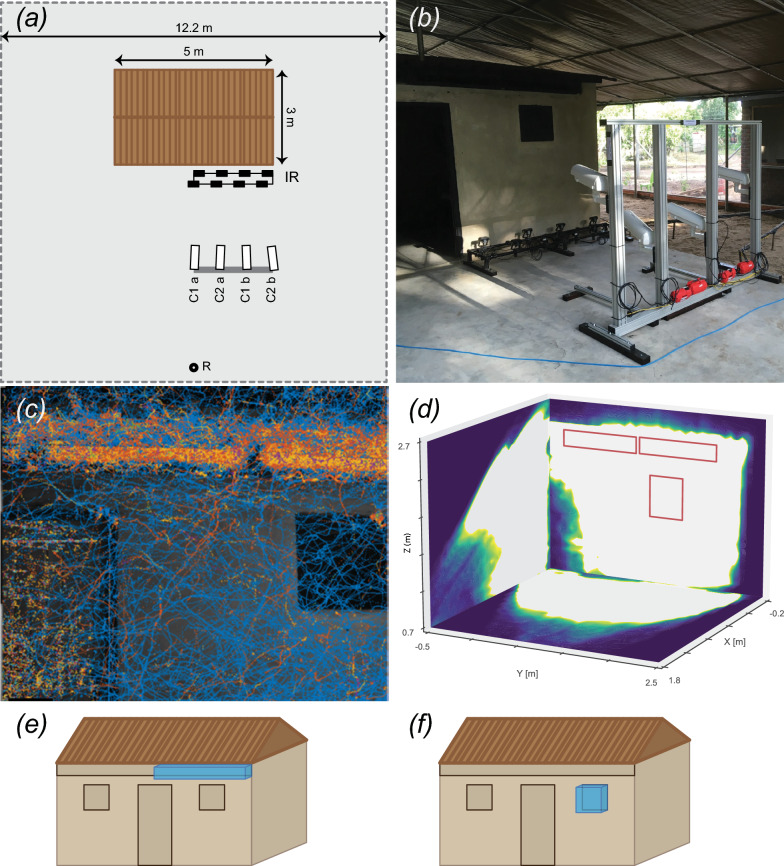
The experimental set-up for studying house-entry behavior of female malaria mosquitoes. **a** Schematic top view of the screened enclosure (12 × 12 m) including the experimental house (brown rectangle 3 × 5 m), the 4 high-speed cameras (labeled C1a, C2a, C1b, C2b), the infra-red lights for camera illumination (IR) and the mosquito release point (R). **b** Picture of the experimental setup, showing the large screened enclosure and within it the experimental house with door, window and metal roof with eave, and the 4 high-speed cameras and infra-red lights. **c** Example showing an overlay of all mosquito flight tracks within 1 experimental night with eaves and windows screened. A blue line was drawn each time a single mosquito entered the view. Orange to red colors are used to indicate when more individuals were tracked at the same time. **d** The three-dimensional (3D) coordinate system and volume in front of the house in which the mosquitoes could be tracked using our videography system. The* X*-axis and* Y*-axis are oriented normal and parallel to the front wall of the house, and the* Z*-axis is oriented vertically. The 3D trackable volume is highlighted in white and projected on the floor, house and the house symmetry plane. The location of the eave and window are indicated in red, with an eave height of between 2.12 and 2.31 m and a window height of between 1.48 and 1.97 m. **e**, **f** To study the flight activity near the eave and window, we defined corresponding volumes-of-interest near these structures, as defined by the blue boxes.

Experiments were performed in a semi-field screened enclosure measuring 12.0 × 12.0 × 2.1–4.0 m (length, width, height) at the Majete Wildlife Park in Chikwawa District, Malawi. The walls of the screened enclosure were made from fiber glass, mosquito-proof screening (Phifer Inc, Tuscaloosa, AL, USA), and the roof was a waterproof tarpaulin. Within this enclosure, we built an experimental house measuring 5.0 × 3.0 × 2.2–2.7 m (length × width × height) (Fig. 1). The walls of the experimental house were constructed from locally produced bricks and plastered with cement, and the roof was made of corrugated iron sheets, including a 20-cm overhang. The front wall of the experimental house was fitted with a door (inner dimensions 197 × 60 cm) in the middle, two windows (30 × 30 cm) and four removable eave frames (inner dimensions 90 × 10 cm per frame). The back wall of the house was also fitted with two windows and four eave frames, but no door. The wooden door, window frames and eave frames were painted black with water-based chalkboard paint.

The windows and eave frames could be left completely open, fitted with insect screens or closed completely with wooden shutters. The screens were made of charcoal-colored fiber glass (Wire Weaving Co. Dinxperlo, The Netherlands), and the shutters were made of plywood painted black with water-based chalkboard paint. Using this system, we were able to systematically investigate the effect of window and eave closure and screening on mosquito house-entry behavior. The door remained closed overnight for all experimental treatments.

Two beds were positioned inside the house, one along each outside wall, and each bed was covered with an untreated bednet. During experimental nights, one adult man slept in each bed, under the bednets, to act as a bait for mosquitoes. Three pairs of adult men volunteered to sleep in the house for 10 experimental nights each. Written informed consent was obtained from the volunteer sleepers. The College of Medicine Research and Ethics Committee (COMREC) in Malawi approved the study (Proposal Number P.02/19/2598). A US Centers for Disease Prevention and Control (CDC) light trap (John W. Hock Ltd, Gainesville FL, USA) was placed near each bed to collect a sample of the mosquitoes that entered the house [28, 29].

### Camera and real-time mosquito tracking set-up

A multi-camera videography system was used to track the three-dimensional (3D) flight kinematics of *An. gambiae* mosquitoes around the experimental house. The videography system consisted of four synchronized machine-vision cameras (Basler acA2040-90umNIR, USB 3.0; Basler AG, Ahrensburg, Germany), equipped with 16-mm f1.4 wide-angle lenses (Kowa LM16HC; Kowa Optical Products Co., Ltd., Nagoya, Japan), with lens aperture set at f2.8. The cameras were operating at 50 frames per second (fps), with a 1-ms exposure time. To improve the light sensitivity of the cameras, pixels within each 2 × 2-megapixel camera image were binned 2 × 2. Binning combines the charge from adjacent pixels (in this case, 2 × 2-pixel bins), resulting in increased light sensitivity but a reduced spatial resolution (in this case, reduced to 1 × 1 megapixel). Image capture on the cameras was synchronized by means of an external trigger pulse, generated by an Arduino Uno microcontroller board (Arduino, Monza, Lombardy, Italy) (https://github.com/strawlab/triggerbox.git).

To protect the cameras and lenses from water, heat and dust, each set was placed in a camera housing (Transpac THP 4000; Basler AG, Ahrensburg, Germany). These camera housings were mounted onto an aluminum frame (MayTec Aluminium Systemtechnik GmbH, Olching, Germany) that was fixed to the concrete floor on which the house was built (Fig. 1b). The cameras were placed at an approximate distance of 2.5 m from the front wall of the house, at heights between 0.8 and 1.3 m. As a result, the camera system imaged the front, right side of the experimental house, including half the door and one window. The cameras were oriented slightly upwards to film the volume below the roof near the eave area. The dimensions of the area in front of the house where mosquitoes could be tracked were approximately 2.5 × 1.0 × 1.5 m (Fig. 1d).

The filming volume was illuminated with eight near-infrared light-emitting-diode (NIR-LED) lights (two ABUS TVAC71000-60° lights and six ABUS TVAC71070-95° lights; ABUS, Volmarstein, Germany). The NIR-LED lights were mounted on a frame placed on the concrete slab directly below the area of interest (Fig. 1b), and the lights were directed upwards and arranged to uniformly light the filming volume near the eave and window, aiming for optimal contrast between the illuminated mosquitoes and the dark background of the house.

We used an automated tracking software [30] to track in real-time the positions of multiple mosquitoes flying in the four camera views, and from these we reconstructed the 3D flight tracks. The tracking software ran on a single laptop (Lenovo ThinkPad P51; Lenovo, Beijing, China) with an Intel Xeon E3-v6 processor and Ubuntu Linux operating system, which performed the real-time image analysis and object tracking for all four cameras, as well as the 3D flight track reconstruction. Based on pilot recordings, sensor gain was set to 1.0 for all cameras, and the maximum number of simultaneously tracked mosquitoes was set to 10. Tracks were reconstructed only when the mosquito was visible in at least two of the four camera views. A dynamic background model was used with update intervals for each 100 frames and a 1% weight factor to compensate for slow changes in illumination conditions.

Cameras were calibrated with the multi camera self-calibration routine [31] by tracking a single moving LED light with each of the four cameras (Cree SunBright 535 nm Green LED; CreeLED Inc, Durham, NC, USA). This calibration was aligned to world reference points based on landmarks on the experimental house. The resulting coordinate system in the world reference frame was defined as X, Y, Z, with the* X*-axis oriented perpendicular to the house front wall, the* Y*-axis oriented parallel to the house front wall along the ground and the* Z*-axis oriented vertically. We defined values within this coordinate system as {*x, y, z*}, with the origin {*x, y, z*} = {0,0,0} located against the house front wall (*x* = 0), on the ground in front of the house (*z* = 0) and (*y* = 0) at the right side of the door frame as observed from the cameras.

The calibration procedure was repeated every experimental day to correct any inadvertent change in camera position. A correction for lens distortions was generated for each camera at the start of the experiment, using a 6 × 10 checkerboard pattern with 90-mm squares. Distortion parameters were computed using openCV procedures (https://docs.opencv.org). Tracking results were corrected for lens distortions.

Videography experiments were performed from 20:00 to 04:00 h. If volunteers briefly left the experimental house during the night, a 5-min buffer period was marked prior to leaving and post re-entering the experimental house. Tracking data within those time slots were removed from further analyses.

### Eave and window modifications

**Fig. 2:**
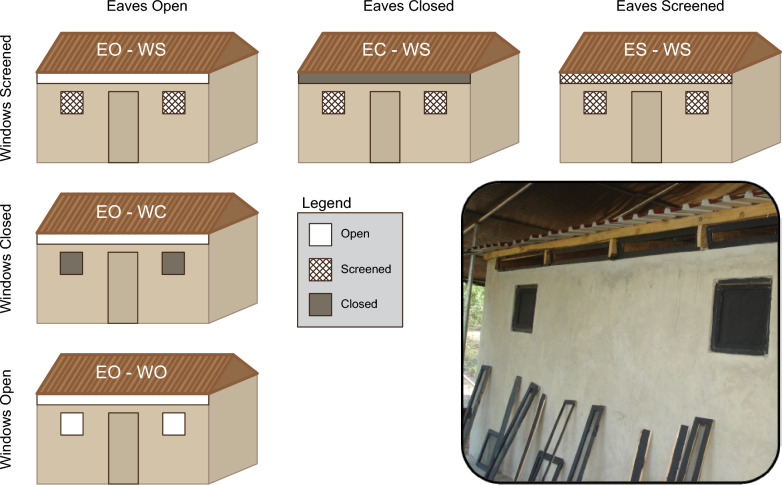
Overview of the five different experimental treatments, in which we systematically closed or screened the window and eave. In the overview, the three rows show the different window treatment conditions (from bottom to top: open, closed and screened), and the three columns show the eave treatments (from left to right: open, closed and screened). Each condition was defined using a four-letter code, where E, W, O, C, and S stand for Eave, Window, Open, Closed and Screened, respectively. The door was closed during all experiments. Eave and window treatments were changed using removable frames, as shown in the inset image. The inset image shows the back of the experimental house, where the eave and window treatments were the same as the front.

We evaluated five experimental house modifications (Fig. 2). For our control condition, both the eaves and windows were fully open (eaves open – windows open [EO-WO]). We used two treatments to test the effect of window modifications on mosquito house entry behavior. In the first treatment, we screened the windows and left the eaves open (EO-WS), and in the second treatment we closed the windows while leaving the eaves open (EO-WC). To test the effect of eave modifications on mosquito house entry behavior, we used treatments in which we screened or closed the eaves while, in each case, screening the windows (ES-WS and EC-WS, respectively).

### Experimental procedure

Before each experiment, the house was prepared by closing, screening or leaving open the eaves and windows, as randomly assigned for each replicate night of the study (Fig. 2). Each treatment was in place for 6 replicate nights (see experimental treatment schedule in Additional file 1: Table S1).

On the day of each experimental replicate, 500 female mosquitoes (5–8 days old and not previously blood-fed), were selected before 12:00 h and set aside in the insectary in a release bucket (diameter 12.5 cm, height 12.5 cm), covered with a mesh and provided with water-soaked cotton wool. Two volunteers slept inside the house under untreated bednets, starting at 19:30 h. The volunteers’ heads were positioned towards the front (door) side of the house, and each pair of volunteers shifted beds (to the left or right side of the house) after each replicate. At 19:30 h, the two CDC light traps at the end of each bed were turned on, with their lights switched off, and the bucket with mosquitoes was placed in the screened enclosure, 5.8 m in front of the experimental house.

At 20:00 h, mosquitoes were released from the bucket by lifting the mesh using a fishing line operated from outside the screened enclosure. Mosquito flight was tracked until 04:00 h, after which the CDC light traps were turned off, and the volunteers could leave the house. Any temporary absence of volunteers during the recording period was recorded in a logbook. A Prokopack aspirator (John W. Hock Company) was used to collect mosquitoes from inside the experimental house at 04:00 h. Together with these Prokopack catches, CDC light trap catches were briefly frozen, and the collected mosquitoes were then counted. Mosquitoes remaining in the release bucket were also counted, and the number of responding mosquitoes for each replicate night was defined by subtracting the number remaining in the release bucket from the initial 500 mosquitoes. Remaining mosquitoes found inside the screened enclosure later that day were removed with the Prokopack and discarded after freezing. Experimental replicates were carried out no more frequently than every other day to ensure proper preparation and to allow any uncaught mosquitoes remaining in the screened enclosure to die before the next experimental replicate.

### Data analysis

The real-time tracking algorithm used a Kalman predictor to reconstruct 3D flight paths from stereoscopic videography data [30], and thus the output data consisted of Kalman-filtered flight paths defined by location, flight velocity and the Kalman covariance error e(*t*). In post-processing, we filtered the resulting database of flight tracks in two steps. First, to remove potential extrapolation errors from the Kalman predictor, we deleted the end of tracks if either the estimated flight speed exceeded 1.5 m/s or the Kalman covariance error was > 0.01. Second, we then discarded all tracks that were shorter than 10 cm or less than 0.2 s (10 video frames at 50 fps). These settings were based on a sensitivity analysis and the assumption that flying *Anopheles* mosquitoes have a maximum flight speed of < 1.5 m/s. The resulting flight paths consisted of the temporal dynamics of the 3D location {*x*(*t*), *y*(*t*),* z*(*t*)} and velocity {*u*(*t*), *v*(*t*),* w*(*t*)} of each flying mosquito; these were used for our subsequent analyses.

We used all combined flight tracks per treatment to calculate average mosquito density distributions and flight velocity distributions around the house. For this, we divided the filming volume into 40 × 40 × 40 voxels (spatial bins), resulting in an approximate voxel size of 5 cm in the X- and Z-direction, and 7.5 cm in the Y-direction. In each voxel we estimated the mosquito density as the relative proportion of time mosquitoes spent in that voxel, defined as T* = *T*_*i*_/*T*_total_, where *T*_*i*_ is the time spent in voxel *i*, and *T*_total_ is the total flight time. We visualized these density distributions as heat maps projected on three two-dimensional (2D) planes (X–Y, X–Z and Y–Z). We determined the flight velocity vector in each voxel as the mean flight velocity of all mosquitoes that passed through that voxel. We visualized the velocity distributions using streamline plots derived from these velocity fields, projected on the same set of 2D planes as for the density distributions (X–Y, X–Z and Y–Z).

For measuring and comparing flight activities near the eave and window area, we defined volumes-of-interest around the eave and window (Fig. 1e, f, respectively). These volumes had the same rectangular or square shape as the eave or window, respectively, but extended 10 cm on each side (in the Y- and Z-direction). The volumes started at the wall and extended 30 cm outward in the direction perpendicular to the wall (in the X-direction). We then identified all flight tracks that intersected these volumes around the eave and window. Based on these, we quantified flight activity around the window and eave using the time that mosquitoes spent in the corresponding volumes. We determined this time spent in each volume by summing all durations that flight tracks remained in the defined volume; this was done for each experimental night and for an array of time bins with a temporal resolution of 10 min.

Next, we used the flight tracks around the window and eave to study when and how mosquitoes visited the window and eave, and when and how they arrived, departed, remained in and returned to these volumes. ‘Arrivals’ were defined as flight tracks that started at least 10 cm outside the volume-of-interest and ended within the volume. ‘Departures’ started within the volume-of-interest and ended at least 10 cm outside the volume. ‘Visitors’ started outside the volume-of-interest, entered the volume, left the volume and finally ended outside the volume. ‘Returnees’ started inside the volume-of-interest, left the volume, re-entered the volume and finally ended inside the volume. ‘Remainers’ started and ended inside the volume-of-interest, without moving outside the volume. It should be noted that if a flight track ended within the window or eave volume, the mosquito might have entered the house or might have landed on the house, because the tracking algorithm only tracked mosquitoes flying outside the house.

Based on these data, we determined the number of mosquitoes that showed each type of flight behavior (visiting, arriving, departing, remaining and returning). We then used the flight kinematics data to determine the behavior-specific flight dynamics around the eave and window. Specifically, we constructed streamline plots, both per treatment and across all 30 replicates, for all mosquitoes that arrived at the volumes around the eave and window. To focus on the approach kinematics only, we removed the parts of the tracks after arrival.

We used analysis of variance (ANOVA) to test for differences among treatments in various flight kinematics and house entry parameters. The dependent parameters were the number of responding mosquitoes, the percentage of responding mosquitoes collected inside the experimental house, flight track duration (time spent) and the number of flight tracks. We used Tukey’s HSD for pairwise comparisons when the ANOVA test showed a significant difference between treatments. We also used ANOVA to test for differences in house entry rates among the three pairs of volunteer sleepers. We defined *P* < 0.05 as significant, 0.05 ≤ *P* < 0.10 as marginal, and *P* ≥ 0.10 as non-significant.

## Results

Experiments were performed on 30 nights (*n* = 6 replicates per treatment) between 16 March and 20 June 2020. The average number of responding mosquitoes, i.e. those that left the release bucket overnight, was 491.1 ± 5.5 per night (98%). There was no effect of house treatment on the number of responding mosquitoes (Additional file 1: Table S2; ANOVA, F = 0.936, *P* = 0.459).

### Indoor mosquito collections

**Fig. 3:**
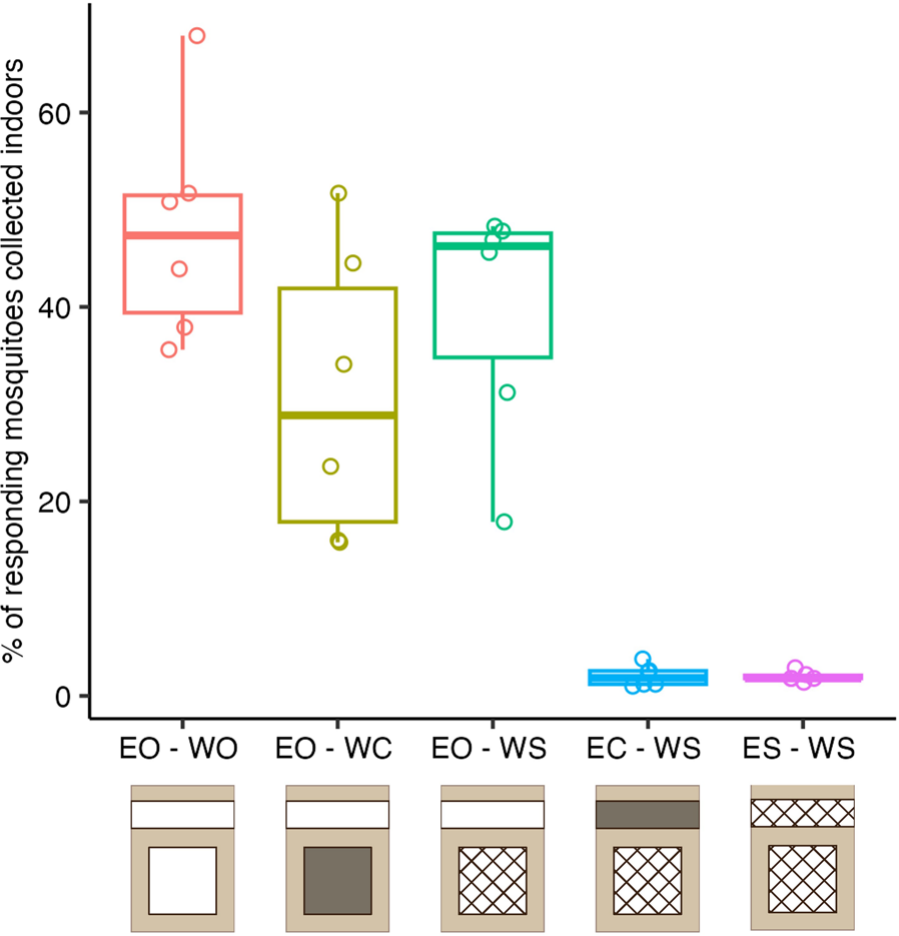
Percentage of responding mosquitoes that were collected indoors at the end of each experiment. Mosquitoes that flew out of the release bucket were marked as “responding,” which ranged from 477 to 499 (out of 500) mosquitoes per replicate. Counts of mosquitoes collected indoors were based on the sum of CDC light trap and Prokopack aspiration. Boxplots show median, 25th and 75th percentiles and fences of collected mosquitoes per night, by treatment (*n* = 6 replicate nights per treatment, indicated by open circles). EO-WO, eaves open – windows open; EO-WC, eaves open – windows closed; EO-WS, eaves open – windows screened; EC-WS, eaves closed – windows screened; ES-WS, eaves screened – window screened

House entry rates based on CDC light trap and indoor Prokopack collections were independent of the three volunteer pairs used to lure the mosquitoes into the experimental house (ANOVA, F = 0.123, *P* = 0.885). The percentage of responding mosquitoes that entered the house was high for all treatments with open eaves, with a median of 47.3% (interquartile range [IQR] 12.1%), 28.9% (IQR 24.0%) and 46.3% (IQR: 12.8%) for the EO-WO, EO-WC and EO-WS treatments, respectively (Fig. 3; Additional file 1: Table S2). Among the three treatments with open eaves, we did not see a significant effect of window treatment (open, closed or screened) on indoor mosquito collections (Fig. 3; Tukey HSD, *P* > 0.05). As expected, among the three treatments with screened windows, closing or screening the eaves drastically reduced the number of mosquitoes found indoors compared to open eaves (Fig. 3; Tukey HSD, *P* < 0.001). The percentage of responding mosquitos caught indoors was reduced to a median of 1.9% (IQR 1.4%) and 1.8% (IQR 0.3%) when eaves were screened or closed, respectively (Fig. 3; Additional file 1: Table S2).

### Mosquito flight activity in space and time

**Fig. 4:**
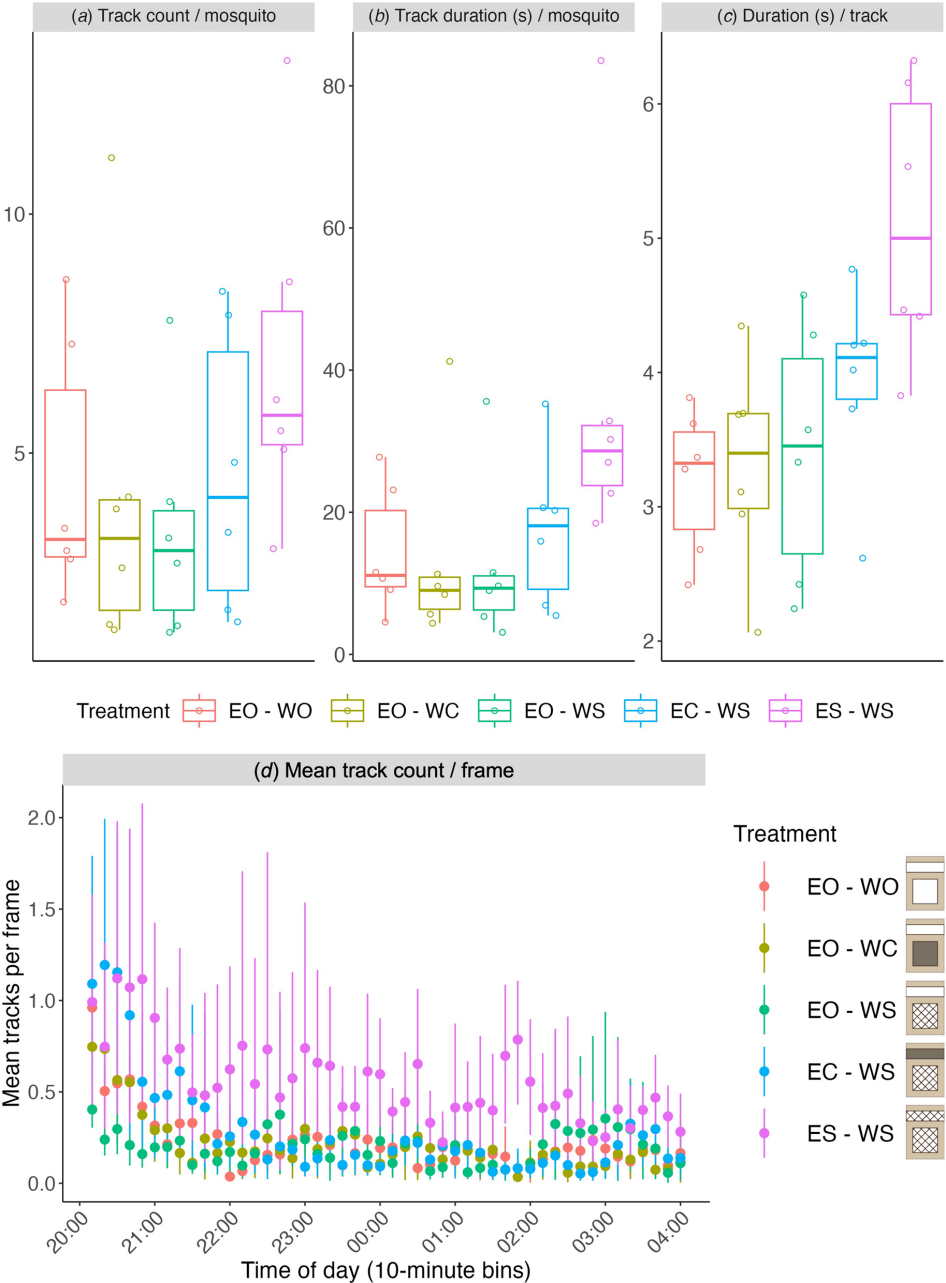
Mean flight activity throughout the night per treatment (**a**–**c**), and the corresponding temporal dynamics of flight activity per treatment (**d**). Data for different treatments are color-coded, as defined at the bottom right panel. **a**–**c** Mean flight activity per treatment was estimated using the mean number of recorded flight tracks per responding mosquito per night (**a**), the mean flight track duration per responding mosquito per night (**b**) and the mean duration per flight track (**c**). **d** The temporal dynamics of flight activity per treatment was estimated using the mean number of observed flight tracks per video frame within 10-min bins. Boxplots in **a**–**c** show the median, 25th and 75th percentiles and fences (*n* = 6 replicate nights per treatment, indicated by open circles). Dots in **d** represent means, and bars show 95% confidence intervals. EO-WO, eaves open – windows open; EO-WC, eaves open – windows closed; EO-WS, eaves open – windows screened; EC-WS, eaves closed – windows screened; ES-WS, eaves screened – windows screened

In total, we recorded and reconstructed 69,025 flight tracks, which resulted in a median of 3.6 (IQR 4.4) flight tracks per responding mosquito per experimental night (Fig. 4a), with a median cumulative track duration per responding mosquito per night of 11.5 s (IQR 17.5 s) (Fig. 4b) and a median track duration per flight of 3.7 s (IQR 1.2 s) (Fig. 4c). The number of simultaneously recorded tracks was well below our tracking algorithm limit of 10 simultaneous tracks (Fig. 4d). The mean number of tracks per video frame varied from approximately one flight track in the first hour of the night (for treatments with eaves screened or closed), to values < 0.5 towards the end of the night, irrespective of treatment (Fig. 4d).

The number of flight tracks per responding mosquito per night (Fig. 4a) was not statistically different between house treatments (ANOVA, F = 1.089, Pr(P) = 0.383). The cumulative track duration per responding mosquito per night (Fig. 4b) was marginally different between treatments (ANOVA, F = 2.582,  Pr(P) = 0.062); the greatest differences in pairwise comparisons were between the treatment with eaves and windows screened (ES-WS) and those with open eaves (EO-WO, EO-WC and EO-WS; Tukey HSD, adjusted *P* = 0.126, 0.099, and 0.077, respectively). Mean duration per track was significantly longer for the house treatment with both eaves and windows screened (ES-WS) than for the treatments with open eaves (EO-WO, EO-WC and EO-WS; Fig. 4c; Tukey HSD, *P* < 0.05), but not for the treatment with eaves closed and windows screened (EC-WS; Fig. 4c; Tukey HSD, *P* = 0.120).

**Fig. 5:**
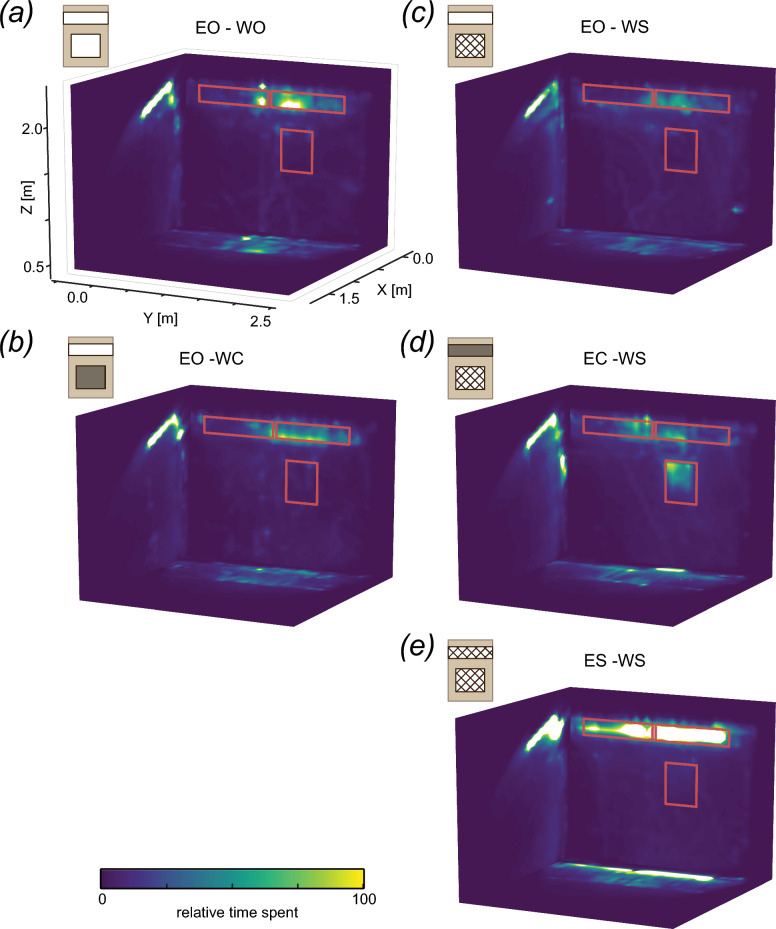
The density distribution of mosquitoes flying in front of the experimental house, for the 5 house treatments. The spatial distribution of relative proportion of time spent in front of the house was estimated in three dimensions. Here, the three-dimensional spatial distribution is projected on to flattened two-dimensional planes for visualization. The X–Y plane shows the flattened distribution as observed from above, looking down to the ground. The Y–Z plane shows the flattened distribution as observed from behind the cameras, looking towards the front surface of the house. The X–Z plane shows the distribution as observed from Y ≥ 2.5 m, looking towards Y = 0 m. The location of the eave and window are indicated using red rectangles on the Y–Z plane. The relative proportion of time spent is shown using the colors indicated by the color bar, where yellow and blue show high and low activity in that area, respectively. EO-WO, eaves open – windows open; EO-WC, eaves open – windows closed; EO-WS, eaves open – windows screened; EC-WS, eaves closed – windows screened; ES-WS, eaves screened – windows screened

**Fig. 6:**
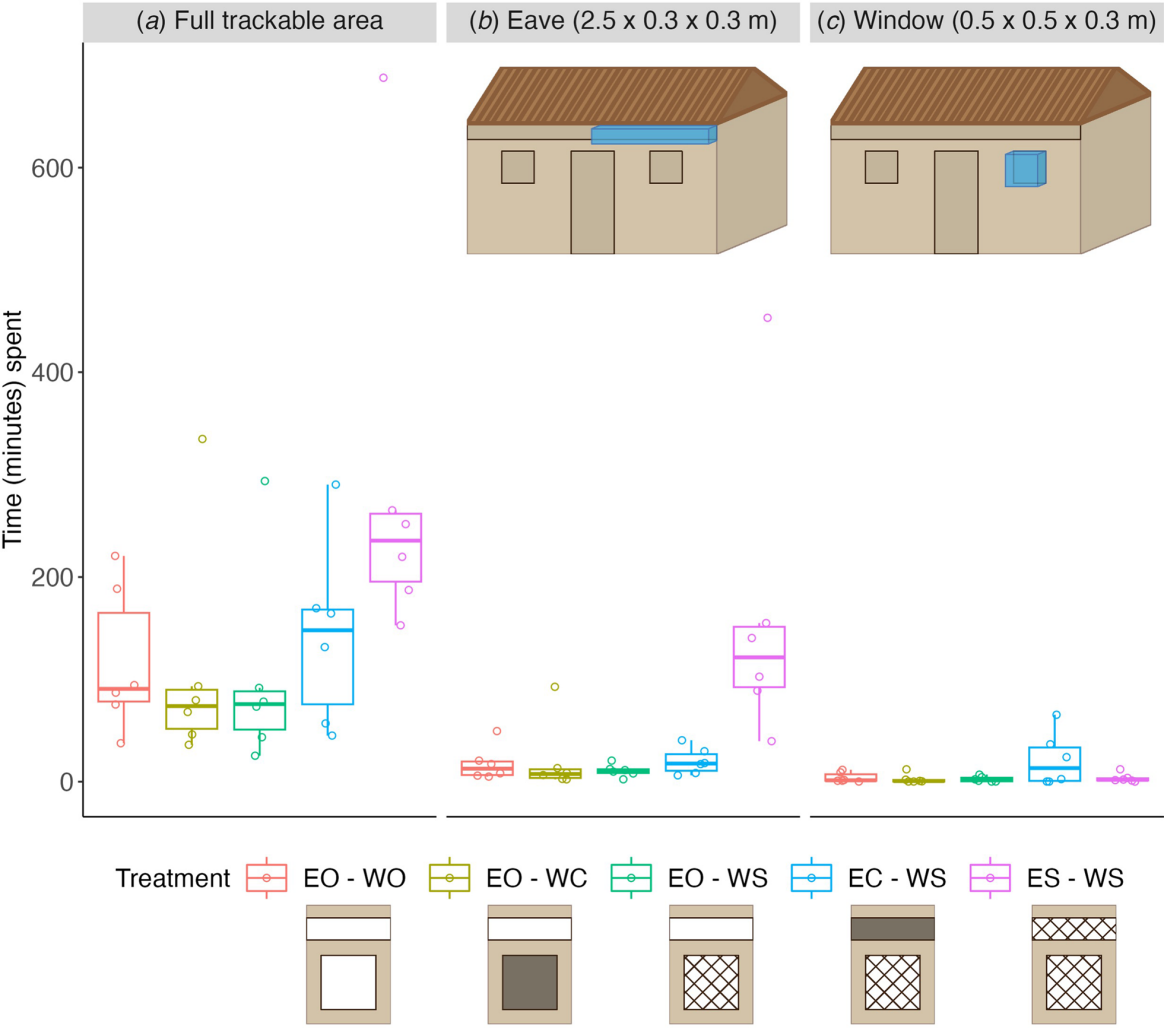
Flight activity of *Anopheles gambiae* in front of the house (**a**), and near the eave (**b**) or window (**c**), separated by treatment. Flight activity was expressed as the time spent in the specified volume over the full night of tracking (20:00–04:00 h). **a** All activity in the trackable area in front of the house (as depicted in Fig. 1d). **b**, **c** Flight activity within the volumes near the eave (**b**) and window (**c**), as indicated by the blue boxes in the house schematics. Results are color-separated by treatment as indicated at the bottom. Boxplots show the median, 25th and 75th percentiles and fences (*n* = 6 replicate nights per treatment, indicated by open circles). EO-WO, eaves open – windows open; EO-WC, eaves open – windows closed; EO-WS, eaves open – windows screened; EC-WS, eaves closed – windows screened; ES-WS, eaves screened – windows screened

The spatial distribution of mosquito flight tracks in front of the experimental house, as measured by the relative proportion of time spent in the 40 × 40 × 40 voxels, was generally concentrated near the eaves of the house (Fig. 5). Based on these spatial distributions of relative proportion of time spent, we estimated the total time spent flying in front of the house by all mosquitoes combined within each treatment (Fig. 6a). The time spent flying in the full trackable area was marginally different between house treatments (Fig. 6a; ANOVA, F = 2.619, P = 0.059). Based on the spatial distributions shown in Fig. 5, the concentration of flight activity near the eaves appeared strongest on the nights with eaves and windows screened (ES-WS; Fig. 5e). Indeed, when we compared the time spent (total track duration) in the volume around the eave, mosquitoes spent significantly more time in the eave area on nights with eaves and windows screened (ES-WS), compared to any other treatment (Fig. 6b; Tukey HSD, *P* < 0.05). Flight activity near the window of the house was generally lower (i.e. less relative proportion of time spent) than near the eaves (Fig. 5). Nights with eaves closed and windows screened (EC-WS) appeared to be an exception (Fig. 5d), with roughly equal amounts of relative proportion of time spent near the windows and eaves on those nights. Indeed, time spent (total track duration) in the defined window area was marginally different among the five treatments (Fig. 6c; ANOVA, F = 2.668, Pr(P) = 0.056), with that for the eaves closed and windows screened (EC-WS) treatment appearing to be higher than those of the other treatments.

**Fig. 7:**
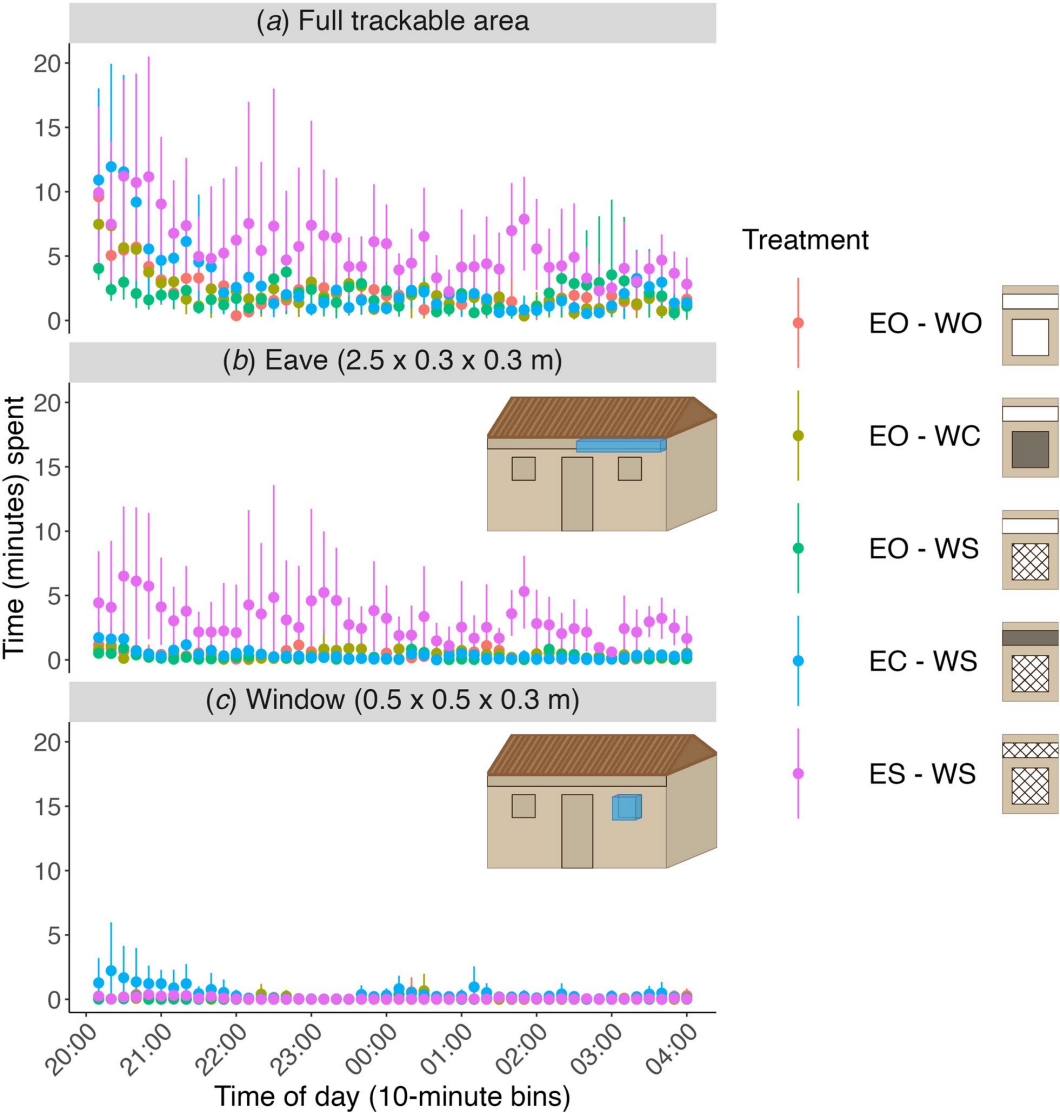
Temporal dynamics of flight activity throughout the night, in front of the house (**a**), and near the eave (**b**) or window (**c**), separated by treatment. Flight activity was quantified as time spent in the specified volume in 10-min bins, from 20:00 to 04:00 h. **a** All flight activity in the trackable area in front of the house. The trackable area is depicted in Fig. 1d. Data in **a** are the same as data in Fig. 4d, but scaled differently for relevant comparisons. **b**, **c** Flight activity in the volumes near the eave (**b**) and window (**c**), as indicated by the blue boxes in the house schematics. Results are color-separated by treatment as indicated on the right and show the mean (dots) and 95% confidence intervals (bars) per treatment (*n* = 6 replicate nights per treatment). EO-WO, eaves open – windows open; EO-WC, eaves open – windows closed; EO-WS, eaves open – windows screened; EC-WS, eaves closed – windows screened; ES-WS, eaves screened – windows screened

Figure 7 shows the time spent overnight in the full recording volume, and near the eave and window, separated in time bins of 10 min. Differences in flight activity among treatments were clearest during the first part of nightly recordings, from 20:00 h when measurements were started to about 21:30 h. During this period flight activity in front of the house was relatively high, and particularly so for treatments with the eave screened or closed (ES-WS and EC-WS, respectively; Fig. 7a). For the treatment with the eave closed (EC-WS), this flight activity rapidly dropped within the first hour, but for the eave screened treatment (ES-WS), this flight activity remained relatively high throughout the night.

Moreover, the location of this increased flight activity differed between these two treatments (ES-WS and EC-WS). For the eave screened treatment(ES-WS), the increased flight activity throughout the night was concentrated in the volume near the eave (Figs. 5, 7b). In contrast, the increased flight activity in the early evening for the closed eave treatment (EC–WS) was mostly concentrated in front of the window (Figs. 5, 7c). For all other treatments, flight activity during the night was much lower and less strongly concentrated in specific areas (Fig. 7), although in all cases the highest flight activity remained near the eave (Fig. 5). Overall, less activity was observed near the window area than the eave area throughout the night (Figs. 5, 7b, c).

**Fig. 8:**
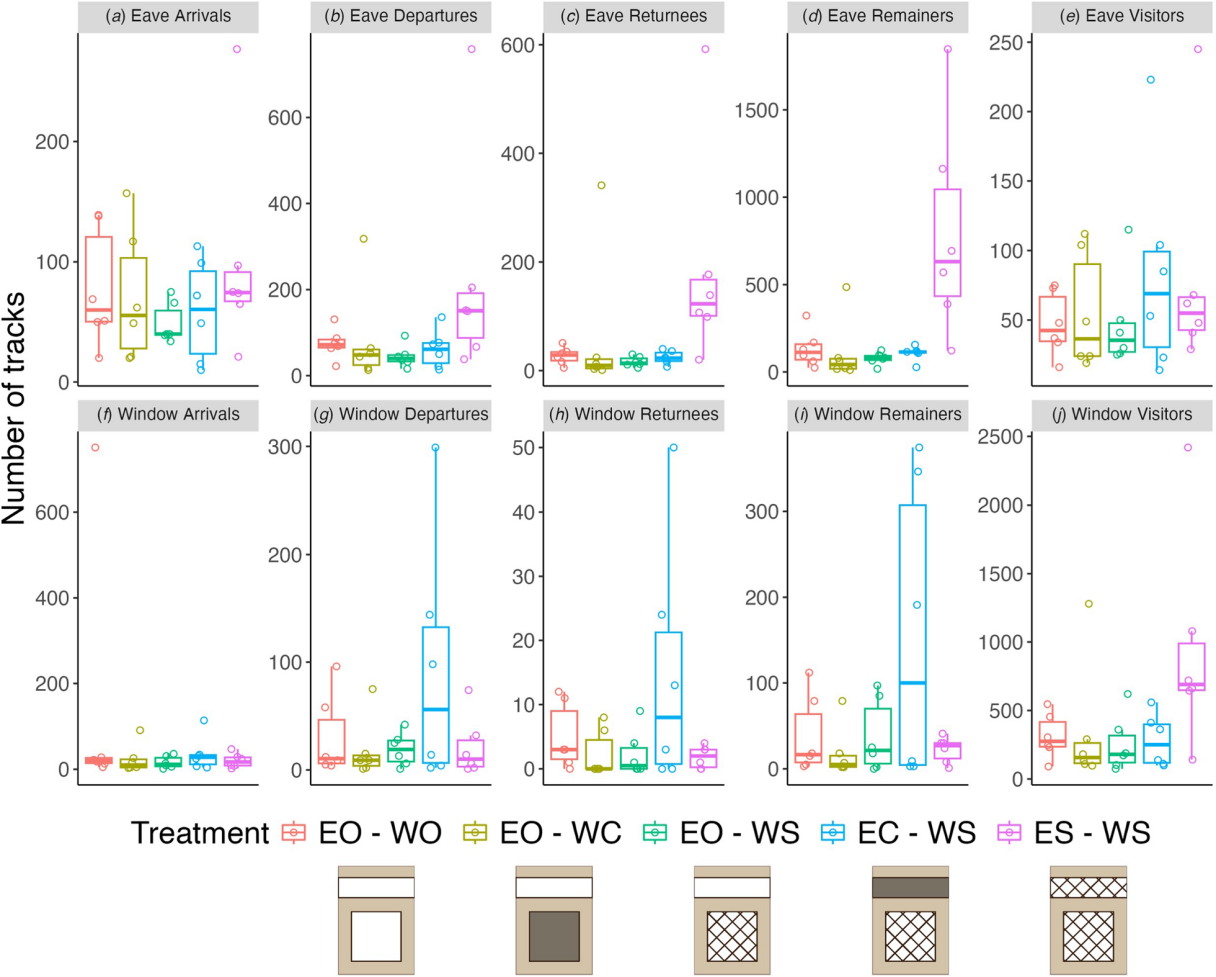
Number of flight tracks of mosquitoes that either arrived, departed, returned, remained or visited the eave-specific (**a**–**e**) or window-specific (**f**–**j**) volumes. The eave-specific or window-specific volumes are defined as shown in Fig. 1e. Results are color-separated by treatment as indicated at the bottom. Boxplots show the median, 25th and 75th percentiles and fences (*n* = 6 replicate nights per treatment, indicated by open circles). EO-WO, eaves open – windows open; EO-WC, eaves open – windows closed; EO-WS, eaves open – windows screened; EC-WS, eaves closed – windows screened; ES-WS, eaves screened – windows screened

We categorized the mosquito tracks according to the flight behavior around the eave and window, for the various house treatments (Fig. 8), characterizing all flights near both the eave and window as “arrivals,” “departures,” “returnees,” “remainers” or “visitors”. The distinct similarities in these categories between the eave and window regions show that the flight behavior around the window and eave is strikingly similar. This is particularly apparent when the behaviors that led to increased activity near the eave and window were compared for the treatments with the eaves screened and closed, respectively (ES-WS and EC-WS in Figs. 5–7). Our behavioral classification shows that the resulting increased flight activity both near the eave (for ES-WS) and near the window (for EC-WS) was largely driven by an increased number of ‘departures,’ ‘returnees,’ and ‘remainers’ for those treatments, compared to the other treatments (Fig. 8). The number of ‘arrivals’ and ‘visitors’ had a smaller effect on the treatment-specific increased flight activity near the eave and window, suggesting that the increased flight activity near the eave and window for the screened and closed eave cases was caused primarily by mosquitoes remaining near, or departing and returning to, those house structures when they were unable to enter the house through the screening, and not by differences between treatments in the initial flight to arrive at these structures.

### Mosquito flight pattern on approaching a house

**Fig. 9:**
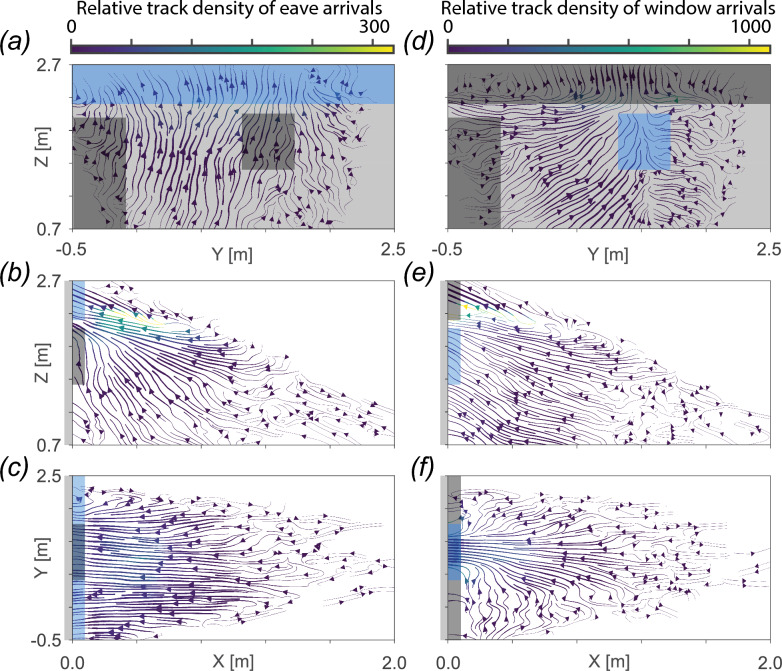
The flight patterns of all mosquitoes approaching the eave (**a**–**c**) and window (**d**–**f**). Results are shown as average streamlines color-coded with relative track density, as defined at the top of each column. Streamline thicknesses show variations in mean flight speed, where thicker lines indicate higher flight speeds. Data are projected on the three planes defined in Fig. 1d: **a**, **d** the house front wall (Y–Z), **b**, **e** the house symmetry plane (X–Z) and **c**, **f** the ground surface (X–Y). The flight patterns are based on all flight tracks of mosquitoes arriving at the eave-specific volume (**a**–**c**) and the window-specific volume (**d**–**f**), as defined in Fig. 1e and shown here in blue. The other house structures, including the house wall, door outline and non-used eave-specific and window-specific volume are shown in gray.

Next, we focused on the approach flights of mosquitoes towards the eave and window (Fig. 9; Additional file 1: Figures S1, S2). Treatment-specific streamline plots of approach flights towards the eave (Additional file 1: Figure S1) and window (Additional file 1: Figure S2) show a consistent approach behavior between the different treatments. We therefore combined all recorded approach flight tracks for the different treatments to reconstruct the average approach flight kinematics for mosquitoes arriving at the eave (Fig. 9a–c) or window (Fig. 9d–f). For both cases, we visualized the average flight pattern of mosquitoes approaching the eave and window using streamlines color-coded with relative track density, and streamline thicknesses defining the flight speed. The streamline data are projected on the three planes defined in Fig. 1d, being the projections on the house front wall (Y–Z), house symmetry plane (X–Z) and ground surface (X–Y).

The results for mosquitoes approaching the eave (Fig. 9a–c) show that, on average, mosquitoes approached the eave using a steady ascending flight, starting at an approximate distance of 1 m from the front wall of the house. In particular, the side view projection shows that the majority of flight tracks (highest density) followed this steady ascending flight directly towards the eave. A second smaller group of mosquitoes (lower density) approached the eave more from below by ascending more steeply once they were closer to the house front wall. This pattern seems independent of treatment (Additional file 1: Figure S1). The streamlines projected on the ground surface (Fig. 9c), reveal that, on average, the mosquitoes approached the house with minimal variation in flight speed parallel to the house (left–right deviations). These results combined show that mosquitoes approaching the eave seem to fly in a highly targeted manner by rapidly ascending in a direct line towards the eave.

The mosquitoes approaching the window showed a similar highly consistent flight pattern (Fig. 9d–f). The top view projection shows that most mosquitoes approached the window in a straight line, starting from relatively far from the house, and that only few mosquitoes approached the window from the sides (Fig. 9f). That said, the side view projection shows that the highest density of flight tracks was located near and directed towards the eave (Fig. 9e), suggesting that many of the mosquitoes arriving at the window flew first towards the eave, after which they turned towards the window. This flight pattern is similar for all treatments (Additional file 1: Figure S2).

**Fig. 10:**
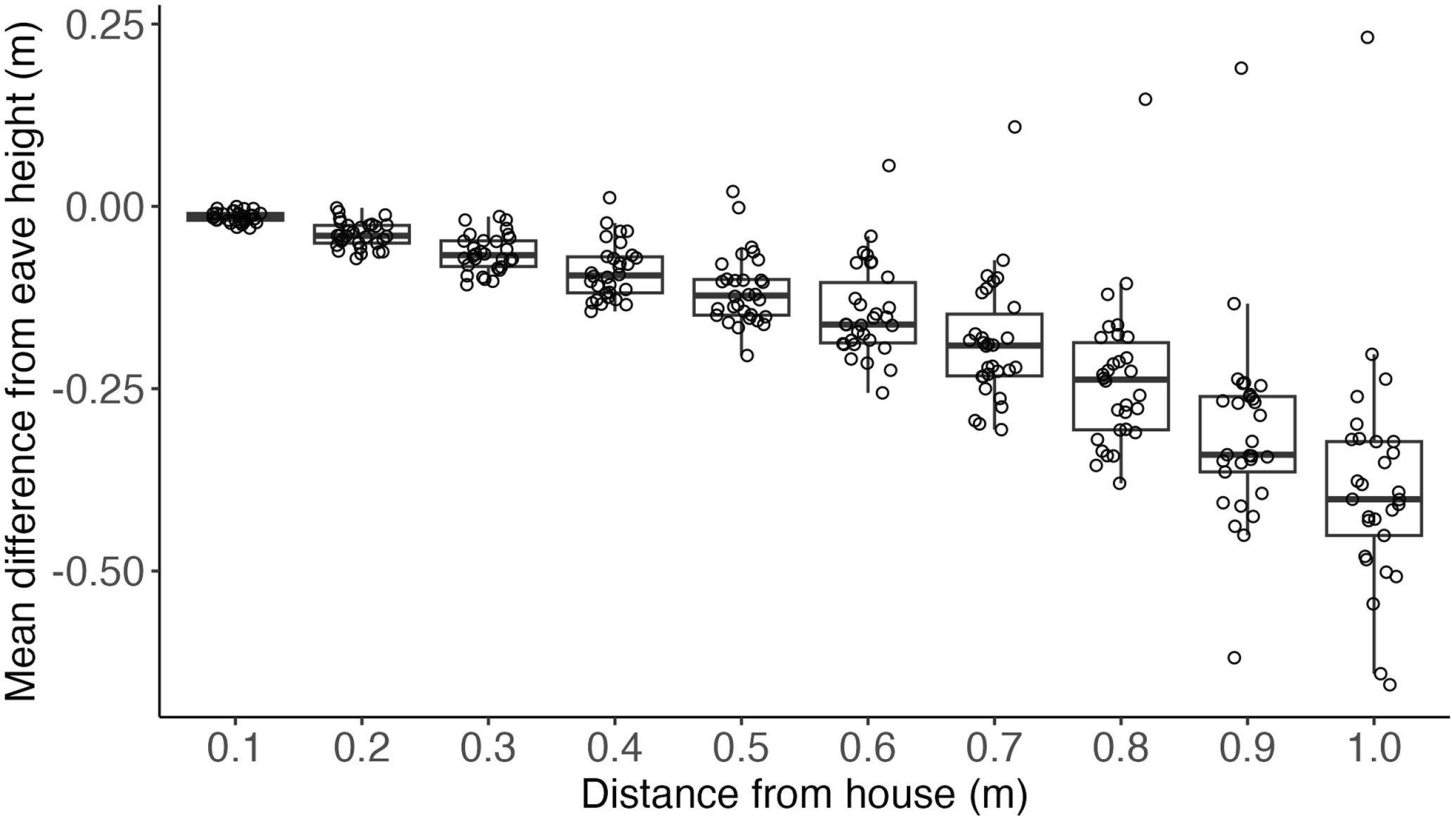
The flight height relative to the height of the eave versus distance from the house, for all mosquitoes approaching the eave. The results are shown as boxplots per distance from the house, at a range of distances from 0.1 to 1.0 m from the house with increments of 0.1 m. Each boxplot shows the median height, the 25th and 75th percentiles and fences (*n* = 30 replicate nights for all treatments combined, indicated by open circles).

The combined density and streamline plots suggest that the majority of flying mosquitoes approached the house by ascending in a direct line towards the eave (Figs. 5, 9). We tested this by estimating flight height (relative to the eave) at various distances from the house, for all mosquitoes that approached the eave (Fig. 10; Additional file 1: Figure S3). This analysis is based on the same dataset as shown in Fig. 9a–c and Additional file 1: Figure S1. On average, the mosquitoes approached the eave by ascending approximately 40 cm over a 1-m distance while flying towards the house, resulting in an average climbing flight angle of 22 degrees during this approach. The increase in flight height with decreasing distance from the house was found to be similar between the five house treatment experiments (Additional file 1: Figure S3), suggesting that the climbing approach flight towards the eave is highly characteristic, and does not change with house modifications, even when the eave is fully closed (EC-WS).

## Discussion

We have studied how host-seeking *An. gambiae* mosquitoes approach and enter a house, and how modifications to common house entry points change these flight behaviors, including persistent attempts to enter when blocked by screening. We tracked free-flying mosquitoes released in a semi-field enclosure, which allowed us to capture the detailed dynamics of *An. gambiae* flight in a large region in front of the house throughout the night. Across all five combinations of eave and window modifications tested in the study, *An. gambiae* approached the house by flying directly towards the eave, in an upward sloping path. When the eaves were open, a large percentage of *An. gambiae* entered the house through the eaves, regardless of whether the windows were open, closed or screened. When the eaves and windows were screened, *An. gambiae* spent more time in the area near the eave, persistently attempting to enter via the eave throughout the night. In contrast, with eaves closed and windows screened, *An. gambiae* spent more time in the area near the window—generally after first approaching the closed eave. Taken together, our results highlight the tendency of *An. gambiae* to direct house entry toward the eaves, and to only divert to other house entry points as a secondary option.

Using our real-time videography-based tracking algorithms, we recorded flight activity over an extended period each night from 20:00 to 4:00 h, which covers the typical activity period of *An. gambiae* [32]. While flight activity continued throughout the night for all house treatments, peak activity occurred near the start of the recording period from 20:00 to 21:00 h. This initial peak in activity, which occurred directly after the mosquitoes were released, is earlier than historically observed in natural settings for this species [32, 33]. We released 500 female *An. gambiae* mosquitoes per experimental night, with about 98% leaving the release bucket, resulting in a median of about 1700 flight tracks per night. It should be noted that some mosquito flights may have been recorded over multiple tracks (e.g. if the mosquito exited and then re-entered the tracking area in front of the house). Still, our data set of nearly 70,000 flight tracks represents about 76 h of mosquito flight in front of an occupied house.

We consistently observed *An. gambiae* approaching the occupied house by flying directly towards the eave along upward sloping flight paths with minimal left–right deviations, irrespective of the eave and window modifications. This characteristic flight pattern was apparent across several methods of visualizing the tracked flights, including the density distribution of all flight tracks (Fig. 5) and the streamline plots of flight tracks categorized as approaching the eave (Fig. 9a–c) and window (Fig. 9d–f). These results align with those of previous studies of *An. gambiae* house entry suggesting an increase in flight altitude to eave level based on indirect observations [12, 14]. Our tracking data show directly that this increase in altitude is initiated at least 1 m from the house for the majority of *An. gambiae*, at a climbing angle of approximately 20 degrees; however, we do not know the point at which these mosquitoes initiate this ascending path, as the mosquito release point was beyond our tracking area. It is likely that these direct, upward sloping flights by *An. gambiae* are specific to their approach trajectory to an occupied house, representing a unique stage of host seeking. Host seeking by *An. gambiae* and other mosquitoes at distances > 10 m from a host (beyond visual range) is thought to consist of zigzag, cast-and-surge flight patterns dependent on wind and habitat factors that determine host odor plume characteristics, based on studies of other insect taxa [34], and supported by wind tunnel experiments in mosquitoes [35, 36]. As mosquitoes move closer to a host, they likely integrate additional sensory cues, including visual and thermal cues, with corresponding changes in flight patterns dependent on the specific mix of cues [9, 37, 38]. Our observations of *An. gambiae* approaching an occupied house are the first direct evidence of their flight patterns at this stage of host seeking, filling a critical knowledge gap considering this species is generally endophilic [3, 4].

Our results confirm that the eave is the most attractive region of human-occupied houses and that the open eave is the primary entry point for *An. gambiae* [17, 39]. Additionally, the behavioral responses to eave modifications confirm that odor cues from the house occupants are important for attracting *An. gambiae* to the eave [10, 40]. When the eave was screened but odor could still exit the eave, mosquitoes continued flying to the eave throughout the night while trying to enter the house there. In contrast, when the eave was fully closed and, thereby, the odor-dispersing airflow was blocked, mosquitoes moved away from the eave and towards the screened window. In this configuration, the window was most likely the primary source of human odor dispersal, causing the mosquitoes to continue to fly there following the initial approach to the eave. This initial approach toward the eave, even when the eave is fully closed, is striking and suggests that other sensory cues apart from odor may be important for approaching the house and eave. Although we did not measure CO_2_ or other host odors, and some odor cues may have been present in the eave area when the eaves were fully closed, the difference between closed and screened eave treatments in mosquito activity near the eaves, with mosquitoes either leaving or persisting in the eave area, respectively, suggests a meaningful difference in the way these treatments were perceived by the mosquitoes. We speculate that *An. gambiae* uses visual cues to orient their initial path to the eaves, potentially guided by a visual contrast where the wall meets the roofline. This is plausible under natural conditions given the capacity of *An. gambiae* to see in light intensities near that of starlight [41] and the estimated visual range of nocturnal mosquitoes to be as much as 5–15 m [42]. In this case, host odor cues, including CO_2_, are likely to be critical for triggering the attraction to visual features [37].

Taken together, the consistent flight pattern of *An. gambiae* when initially approaching the house and the divergent subsequent behaviors of these mosquitoes in response to either screened eaves or closed eaves provide guidance for the optimum placement of vector control tools on or near houses, such as eave tubes [24], odor-baited traps [43–45] or push–pull strategies [25–27]. The low amount of flight activity at ground level near the house suggests that placing odor-baited traps or other attractant-based interventions at such locations would be less effective than placing them closer to the eave or farther from the house. The persistence of *An. gambiae* to attempt house entry near screened, but not closed, eaves suggests that placing odor-baited traps near screened eaves would be more effective than placing them near closed eaves. However, the effectiveness of odor-baited traps would also depend on the attractiveness of the trap relative to competing attractants and the capture efficiency of the trap [46], warranting further studies with specific odor-baited traps to determine the optimal location for maximum catch rates.

Screening and closing the window while leaving the eave open had a strikingly small effect on *An. gambiae* flight behavior and did not reduce house entries. When the eaves were completely closed, we detected an increased flight activity around the screened windows, but these were mostly secondary approaches after the mosquitoes had visited the closed eave (Fig. 9e). These results confirm that window modifications such as screens or shutters are ineffective vector control tools for houses with open eaves if not paired with eave modifications, as previously shown [17].

## Conclusions

The results of this study provide the first direct evidence that female *An. gambiae* approach a house using a characteristic flight pattern, flying directly towards the house eave along a climbing flight path. Preventing house entry with screened eaves resulted in prolonged flight activity near the eave as mosquitoes continued to attempt entry at this same point. When the eave was fully closed, presumably preventing host odors from accumulating in the eave area, mosquitoes were deflected to the screened window after the initial approach to the eave. These divergent behaviors of *An. gambiae* after approaching screened and closed eaves may provide guidance for effective positioning of odor-baited traps or other outdoor vector control tools to remove mosquitoes from the population. Further studies on how mosquitoes approach and enter houses could build on our findings by incorporating additional house designs, for example increasing ventilation or the presence of indoor lights [40, 47].

## Supplementary Information

Additional file 1:Table S1. Experimental treatment schedule. Table S2. Indoor mosquito sampling. Figure S1. The average flight patterns of mosquitoes approaching the eave, separated per treatment. Figure S2. The average flight patterns of the mosquitoes approaching the window, separated per treatment. Figure S3. The flight height relative to the height of the eave at various distances from the house and different treatments, for all mosquitoes approaching the eave.

## Data Availability

Experimental data that supports the findings of this study are available in an online repository hosted by the Open Science Framework (OSF) (https://osf.io/y89cx/).

## Abbreviations

EC-WS: Eave closed – window screened
EO-WC: Eave open – window closed
EO-WO: Eave open – window open
EO-WS: Eave open – window screened
ES-WS: Eave screened – window screened
