# Supplementary material for: The effect of eave and window modifications on house entry behavior of Anopheles gambiae
Source: Parasit Vectors. 2025 Jul 3;18:251. doi: 10.1186/s13071-025-06887-9 (PMC12231866; doi:10.1186/s13071-025-06887-9)
Supplement: Supplementary file 1 — Additional file 1:Table S1. Experimental treatment schedule. Table S2. Indoor mosquito sampling. Figure S1. The average flight patterns of mosquitoes approaching the eave, separated per treatment. Figure S2. The average flight patterns of the mosquitoes approaching the window, separated per treatment. Figure S3. The flight height relative to the height of the eave at various distances from the house and different treatments, for all mosquitoes approaching the eave. [file 13071_2025_6887_MOESM1_ESM.pdf]

Supplementary Information

The effect of eave and window modifications on house entry behavior of *Anopheles gambiae*

Spitzen *et al.*

Table S1. Experimental treatment schedule. EO-WO: eave open-window open; EO-WS eave open-window screened; EO-WC eave open-window closed; ES-WS eave screened-window screened; EC-WS eave closed-window screened.

Night	Treatment	Volunteer Pair
1	ES - WS	B
2	EO - WS	A
3	EO - WC	B
4	EO - WO	B
5	EC - WS	B
6	EO - WS	B
7	EO - WO	A
8	EO - WC	A
9	EC - WS	A
10	ES - WS	A
11	EO - WO	C
12	EO - WC	C
13	ES - WS	C
14	EC - WS	C
15	EO - WS	C
16	EO - WS	B
17	ES - WS	B
18	EC - WS	B
19	EO - WC	B
20	EO - WO	B
21	EO - WC	A
22	EO - WS	A
23	ES - WS	A
24	EC - WS	A
25	EO - WO	A
26	EO - WS	C
27	EC - WS	C
28	ES - WS	C
29	EO - WC	C
30	EO - WO	C

Table S2. Indoor mosquito sampling. The number of mosquitoes collected indoors by Prokopack aspiration and CDC light trap are shown by treatment (n=6 replicate nights per treatment). Total collected is the sum of the two methods. Mosquitoes that flew out of the release bucket were marked as “responding”, which ranged from 477 to 499 (out of 500) per replicate. EO-WO: eave open-window open; EO-WS eave open-window screened; EO-WC eave open-window closed; ES-WS eave screened-window screened; EC-WS eave closed-window screened.

	EO - WO Median (IQR)	EO - WC Median (IQR)	EO - WS Median (IQR)	EC - WS Median (IQR)	ES - WS Median (IQR)
Indoor Prokopack aspiration	154.0 (43.3)	98.5 (90.8)	165.0 (47.0)	5.5 (5.3)	5.5 (1.0)
Indoor CDC light trap	79.0 (16.5)	44.0 (27.8)	63.0 (19.3)	2.5 (3.3)	3.5 (1.8)
Total collected indoors	233.0 (62.8)	142.5 (119.3)	228.5 (67.3)	9.0 (6.8)	9.0 (1.5)
Total responding mosquitoes	490.0 (6.5)	491.5 (7.5)	489.5 (3.3)	494.5 (1.8)	495.0 (7.3)
Percent responding collected indoors	47.3% (12.1)	28.9% (24.0)	46.3% (12.8)	1.9% (1.4)	1.8% (0.3)

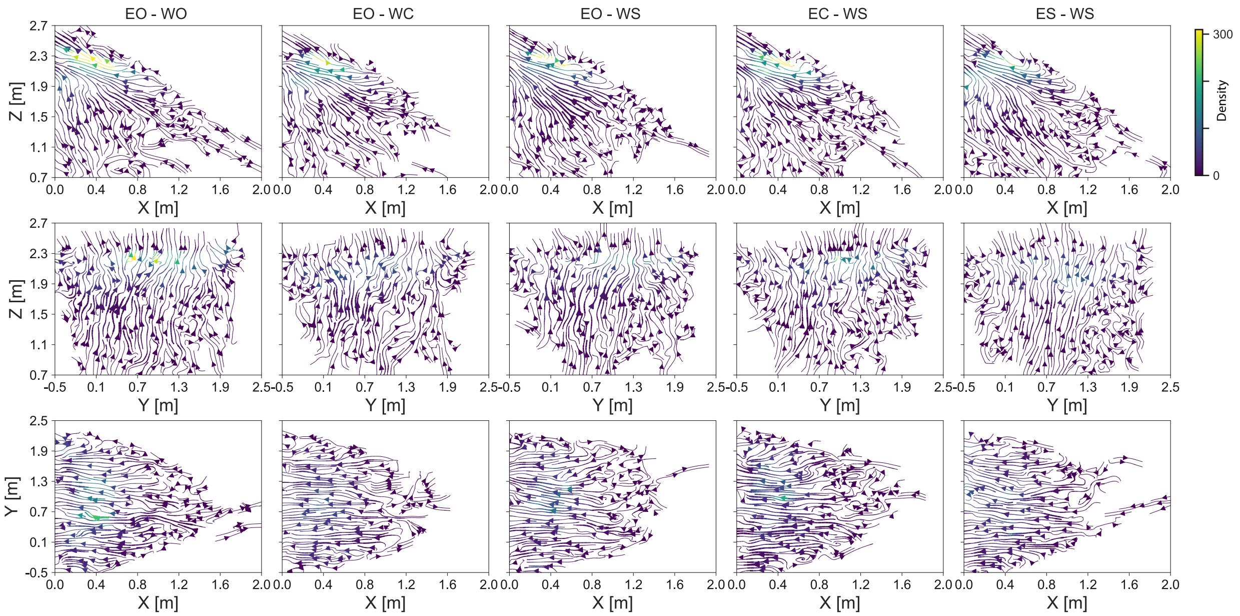

Figure S1. The average flight patterns of mosquitoes approaching the eave, separated per treatment. Results are based on all flight tracks that arrived at the eave-specific volume (as defined in figure 1e), excluding the parts of the tracks after entering the volume. These arrival tracks are equal to the “eave arrivals” in figure 7. The flight patterns are visualized as treatment-specific average streamlines, color-coded with mean flight speed (see color-bar), and projected on the three planes defined in figure 1d. The five columns show results for the different treatments (see codes on top); the top to bottom rows show the projections on the house symmetry plane (X-Z), house front wall (Y-Z), and ground surface (X-Y), respectively.

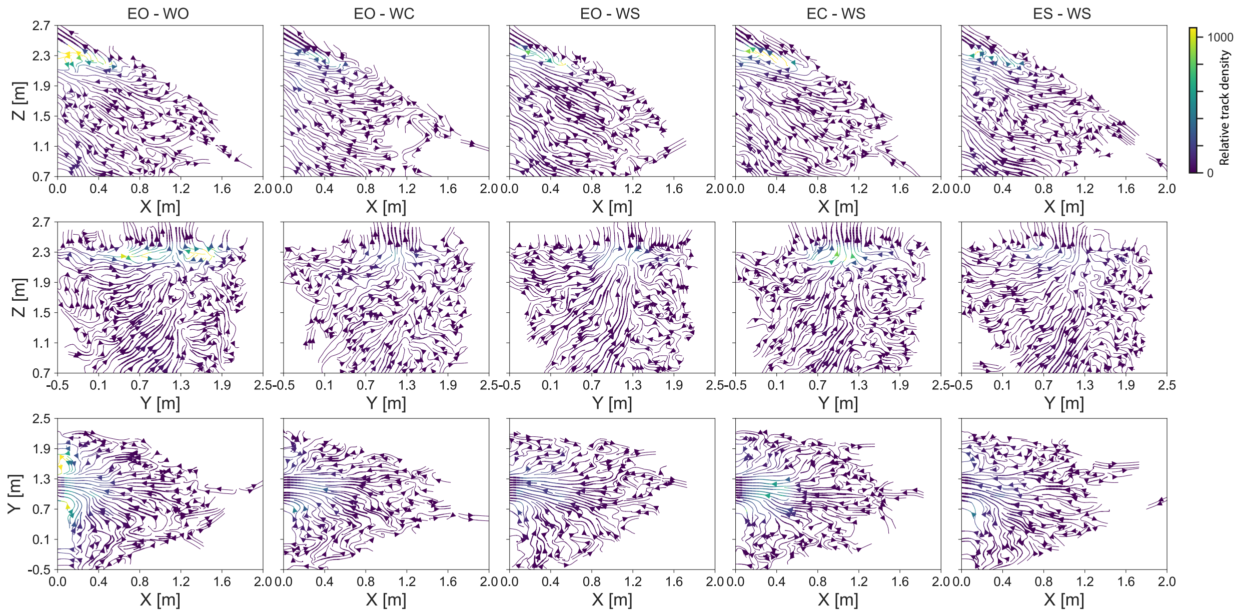

Figure S2. The average flight patterns of the mosquitoes approaching the window, separated per treatment. Results are based on all flight tracks that arrived at the window-specific volume (as defined in figure 1e), excluding the parts of the tracks after entering the volume. These arrival tracks are equal to the "window arrivals" in figure 7. The flight patterns are visualized as treatment-specific average streamlines, color-coded with mean flight speed (see color-bar), and projected on the three planes defined in figure 1d. The five columns show results for the different treatments (see codes on top); the top to bottom rows show the projections on the house symmetry plane (X-Z), house front wall (Y-Z), and ground surface (X-Y), respectively.

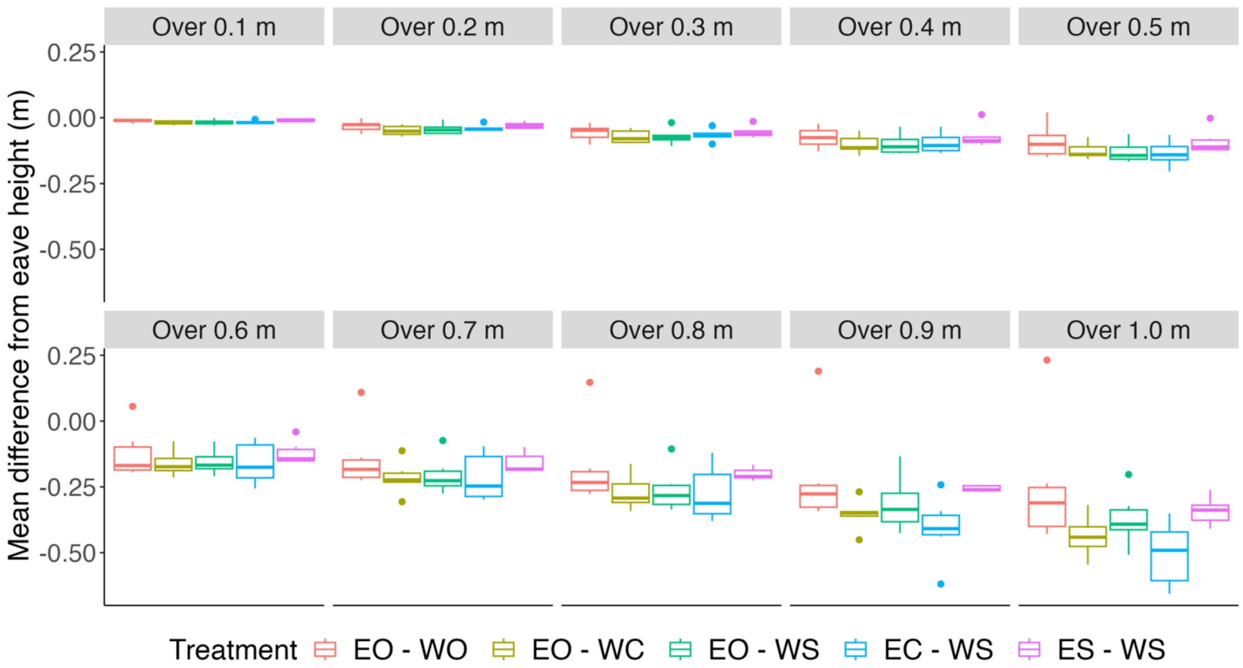

Figure S3. The flight height relative to the height of the eave at various distances from the house and different treatments, for all mosquitoes approaching the eave. The results are shown as boxplots of relative height per treatment (color-coded as defined on the bottom), and per distance from the house (panel titles). The distances range from 0.1 m to 1.0 m from the house with increments of 0.1 m. Each boxplot shows the median height, the 25th and 75th percentiles, fences and outliers (*n*=6 replicate nights per treatment).
